# Diagnostic Value of CT- and MRI-Based Texture Analysis and Imaging Findings for Grading Cartilaginous Tumors in Long Bones

**DOI:** 10.3389/fonc.2021.700204

**Published:** 2021-10-14

**Authors:** Xue-Ying Deng, Hai-Yan Chen, Jie-Ni Yu, Xiu-Liang Zhu, Jie-Yu Chen, Guo-Liang Shao, Ri-Sheng Yu

**Affiliations:** ^1^ Department of Radiology, Cancer Hospital of the University of Chinese Academy of Sciences (Zhejiang Cancer Hospital), Hangzhou, China; ^2^ Institue of Cancer and Basic Medicine (ICBM), Chinese Academy of Sciences, Hangzhou, China; ^3^ Department of Radiology, Second Affiliated Hospital, Zhejiang University School of Medicine, Hangzhou, China

**Keywords:** cartilaginous tumors, texture analysis, chondrosarcoma, enchondroma, radiology

## Abstract

**Objective:**

To confirm the diagnostic performance of computed tomography (CT)-based texture analysis (CTTA) and magnetic resonance imaging (MRI)-based texture analysis for grading cartilaginous tumors in long bones and to compare these findings to radiological features.

**Materials and Methods:**

Twenty-nine patients with enchondromas, 20 with low-grade chondrosarcomas and 16 with high-grade chondrosarcomas were included retrospectively. Clinical and radiological information and 9 histogram features extracted from CT, T1WI, and T2WI were evaluated. Binary logistic regression analysis was performed to determine predictive factors for grading cartilaginous tumors and to establish diagnostic models. Another 26 patients were included to validate each model. Receiver operating characteristic (ROC) curves were generated, and accuracy rate, sensitivity, specificity and positive/negative predictive values (PPV/NPV) were calculated.

**Results:**

On imaging, endosteal scalloping, cortical destruction and calcification shape were predictive for grading cartilaginous tumors. For texture analysis, variance, mean, perc.01%, perc.10%, perc.99% and kurtosis were extracted after multivariate analysis. To differentiate benign cartilaginous tumors from low-grade chondrosarcomas, the imaging features model reached the highest accuracy rate (83.7%) and AUC (0.841), with a sensitivity of 75% and specificity of 93.1%. The CTTA feature model best distinguished low-grade and high-grade chondrosarcomas, with accuracies of 71.9%, and 80% in the training and validation groups, respectively; T1-TA and T2-TA could not distinguish them well. We found that the imaging feature model best differentiated benign and malignant cartilaginous tumors, with an accuracy rate of 89.2%, followed by the T1-TA feature model (80.4%).

**Conclusions:**

The imaging feature model and CTTA- or MRI-based texture analysis have the potential to differentiate cartilaginous tumors in long bones by grade. MRI-based texture analysis failed to grade chondrosarcomas.

## Introduction

Cartilaginous neoplasms are a heterogeneous group of bone tumors with abundant chondroid matrix and hyaline cartilage differentiation (1). The majority of cartilaginous neoplasms are enchondromas, the second most common benign bone tumors, with an incidence of 2.9% in the knee and 2.1% in the shoulder as detected by routine MR examinations (2). Chondrosarcomas are the second most frequent malignant bone tumors next to osteosarcoma, accounting for nearly 20–27% of bone sarcomas (3), and can be stratified into grades 1 to 3 and dedifferentiated chondrosarcoma based on their histopathological findings (1, 4).

Notably, 30% of chondrosarcomas are grade 1, which are low-grade neoplasms with low recurrence rates, locally aggressive behavior and limited metastatic probability (5, 6). In contrast, high-grade chondrosarcomas include grade 2, grade 3, and dedifferentiated chondrosarcomas, and these have higher recurrence rates, metastatic spread and poor survival outcomes, with a five-year survival rate of 53% (7). Given the indolent clinical course of enchondromas, active surveillance is supported to avoid unnecessary surgeries; meanwhile, surgical excision is of paramount importance for chondrosarcomas (8, 9). On imaging, typical chondrogenic tumors present with lobulated patterns, with hyperintensity on T2-weighted magnetic resonance imaging (MRI) and ring- and arc- or popcorn-like calcifications on computed tomography (CT) (10). However, enchondromas and low-grade chondrosarcomas can both demonstrate typical chondrogenic images. Therefore, the overlap of radiological features and histopathological criteria between benign enchondromas and low-grade chondrosarcomas as well as between low-grade and high-grade chondrosarcomas has led to difficulty in correctly and reliably differentiating and grading cartilaginous tumors; thus, a more accurate method is needed.

As a novel tool for objective quantitative assessment of the heterogeneity of lesions, texture analysis can extract, analyze, and interpret imaging features and has been widely used in differential diagnosis, grading, tumor staging and therapeutic response (11–13). Radiomics nomogram based on non-enhanced MRI showed hopeful performance in distinguishing enchondroma from chondrosarcomas, but their performance in differentiating high-grade chondrosarcomas from low-grade chondrosarcomas has not been sufficiently proven (14). CT has been less commonly used to grade cartilaginous tumors than MRI, and no study based on CT texture analysis has been report (15). However, MRI has been applied to analyze cartilaginous tumors but is less effective for visualizing calcification or bone destruction than CT (16, 17). Thus, we evaluated the radiological characteristics through both CT and MRI and extracted texture features. Therefore, this study aimed to assess and validate the diagnostic performance of CT-based texture analysis (CTTA) and MRI-based texture analysis for grading cartilaginous tumors in long bones and to compare these findings to radiological features.

## Materials and Methods

This retrospective study was approved by our institutional review board, and the requirement for informed consent was waived.

### Patients

Patients with long bone cartilaginous tumors confirmed by pathology at the Second Affiliated Hospital, Zhejiang University School of Medicine from January 1, 2009, to May 1, 2015 were retrospectively recorded as the training group. The inclusion criteria were as follows: 1) detailed pathological information, especially tumor grade; 2) CT and MRI examinations at a maximum time interval of 2 months before surgery or biopsy; and 3) enchondromas and chondrosarcomas were found in long bones, including the humerus, radius, ulna, femur, tibia, and fibula. The exclusion criteria were as follows: 1) no intergraded imaging data of MRI or CT for evaluation (n=6) and 2) an unclear pathology report (n=3). Finally, 65 patients with cartilaginous tumors were included in our study, including 29 patients with enchondromas, 20 with low-grade chondrosarcomas, and 16 with high-grade chondrosarcomas. Among these patients, all underwent plain CT scans, while 40 underwent contrast-enhanced MRI examinations, including 17 patients with enchondromas, 14 with low-grade chondrosarcomas and 9 with high-grade chondrosarcomas; the other patients had plain MRI scans only. The average time interval between CT/MRI and surgery or biopsy was 7.4/9.2 days, ranging from 0 to 52 days.

### Imaging Acquisition

A variety of MRI machines (SIEMENS Aera 1.5T; SIEMENS Avanto 1.5T; GE Healthcare Signa HDxt 1.5T) was used due to our retrospective design, and different coils based on different lesion locations were used. However, standard pulse sequences including fat-suppressed T2-weighted imaging (T2WI; repetition time (TR), 2300–6600 ms; echo time (TE), 25–110 ms), axial T1-weighted imaging (T1WI; TR, 190–600 ms; TE, 1.2–12 ms), and fat-suppressed contrast-enhanced T1WI (TR, 420–630 ms; TE, 7–18 ms) were performed on each patient. The field of view (FOV) varied from 120 x 120 mm to 380 x 380 mm because of the different locations, the matrix size ranged from 288 x 224 to 320 x 224, and the slice thickness was 3 mm or 5 mm. Gadolinium-diethylenetriamine pentaacetic acid (Gd-DTPA, Omniscan, 0.1 mmol/kg body weight, 0.5 mmol/ml) was intravenously administered at a rate of 2 ml/s. Furthermore, longitudinal imaging (coronal or sagittal) was acquired for each patient.

Two kinds of CT scanners (Siemens Emotion 64 and TOSHIBA Aquilion 16) were used. Patients underwent plain CT scans, and the parameters were as follows: voltage, 120 kV; maximum tube current, 250 mAs; slice thickness, 3 mm; reconstructed slice thickness, 1 mm; slice collimation, 0.6 mm; and FOV, 380 X 380 or 120 x 120 mm.

### Imaging Analysis

Two musculoskeletal radiologists (with 15 and 8 years of clinical experience in musculoskeletal radiology) reviewed the imaging features without knowledge of the pathological outcomes. Disagreements were settled by consensus after discussion with a third radiologist with 32 years of experience in radiology.

Demographic information, including age, sex, and symptoms, was included. Imaging features were collected as follows: 1) the largest diameter, which was evaluated by measuring the maximum tumor extent in centimeters in axial scans; 2) the aspect ratio was defined as the length divided by width of the lesion, which accounted for the anisotropy of the shape of the tumor; 3) the location was defined as epiphysis, metaphysis, and diaphysis; 4) the calcified shape was defined as ring or plaque calcification and ground glass calcification in CT; 5) the calcified area was defined as the ratio of calcification to the tumor diameter at the maximum diameter plane and was stratified into<1/3, 1/3~2/3, and>2/3; 6) endosteal scalloping was defined as a local thinning of the osseous cortex by the nearby lesions, which formed a lobular outline (4); 7) periosteal reaction was defined as abnormal thickness or focal proliferation of the periosteal; 8) cortical destruction was defined as a sclerotic or lytic process destroying the continuity of the cortical bone; 9) blurring edge was characterized as an ill-defined tumor margin; 10) Fat replacement, which meant there was a fat-like signal surrounded by tumor tissue; 11) hemorrhage was defined as the presence of a bleeding signal in MRI, which was high signal intensity on T1WI, and low signal on T2WI; 12) peritumoral edema, which was defined as high signal intensity on fat-suppressed T2WI surrounding soft tissue without contrast enhancement; 13) soft tissue mass was defined as normal tissue that was replaced or displaced by a solid, extraosseous mass with contrast enhancement; and 14) the contrast enhanced pattern, which was evaluated on MRI and was divided into three types—type I was defined as a mostly continuous ring- and arc-enhancement of the lesion, type II was defined as a small patchy enhancement with or without ring- and arc- enhancement, and type III was defined as a large patchy, nodule enhancement or with obvious enhancement of the internal septa thickness.

### Texture Analysis

MaZda software was used to extract features from CT, T1WI, and T2WI data. First, imaging intensity was normalized and standardized to rectify the effect of different imaging protocols (18). Nine histogram features were extracted in this study, including the mean, variance, skewness, kurtosis, perc.01%, perc.10%, perc.50%, perc.90%, and perc.99% (19). The region of interest (ROI) was outlined in the slice with the maximum diameter of the tumor without any artifacts.

### Validation Group

**Figure 1:**
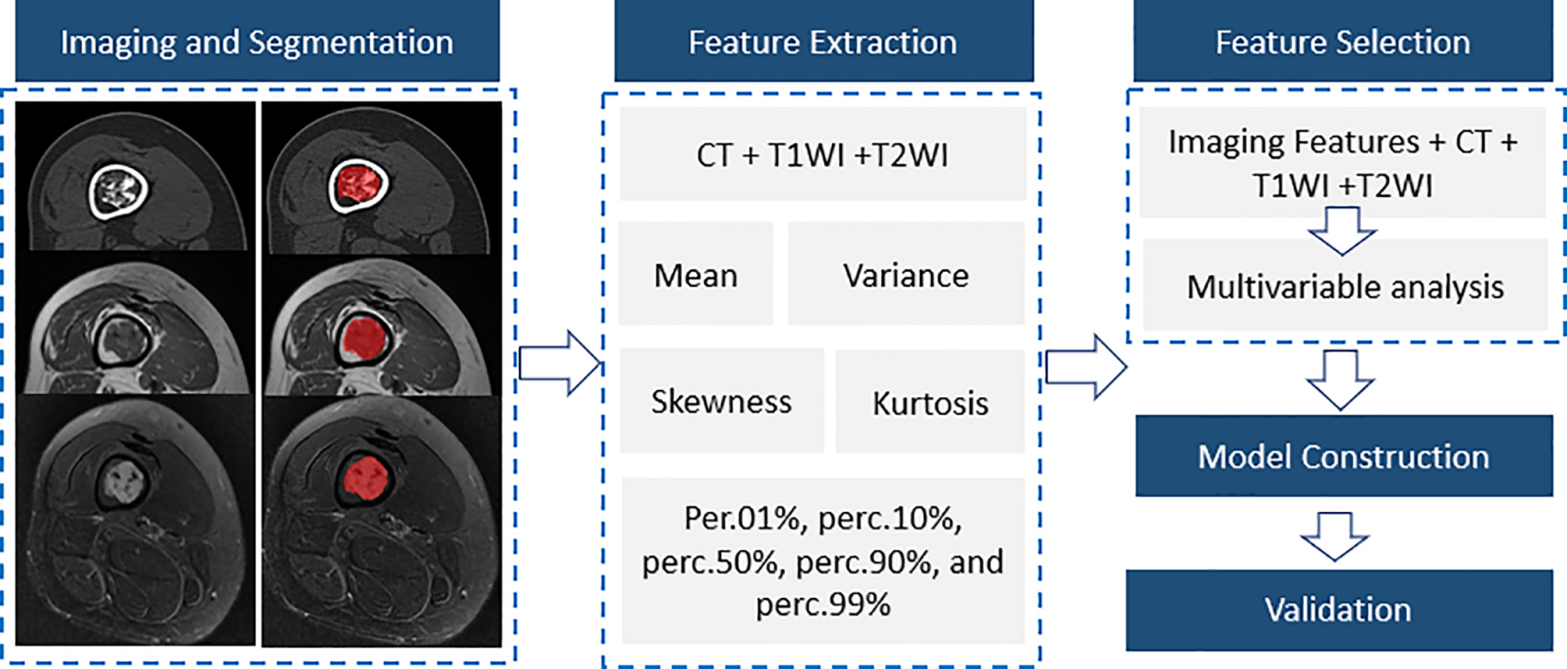
A flow diagram of the whole study including imaging and segmentation, feature extraction, feature selection, model construction and validation.

The training and validation group was set at a ratio of 5:2. Thus, another 26 patients with long bone cartilaginous tumors confirmed by pathology at the same center from January 1, 2016 to August 1, 2021 were retrospectively recorded as the validation group. Only significant variables from the training group were evaluated to verify the performance of different models. As shown in **Figure 1**, our study consisted of five steps, including imaging and segmentation, feature extraction, feature selection, model construction and validation.

### Statistical Analysis

According to the data distributions, continuous data are presented as the means ± standard deviation or medians (25–75%), while qualitative data are expressed as the frequencies (%). Student’s t-tests or Mann-Whitney U tests were used for quantitative data, while Fisher’s exact tests or χ2 tests were used for categorical data. Moreover, binary logistic regression analysis was performed for variables (p values less than 0.05 in the previous analysis) following the stepwise backwards procedure to differentiate cartilaginous tumors in different models. Then, receiver operating characteristic (ROC) curves were calculated to determine the diagnostic capacity of the model, and the accuracy rate, sensitivity, specificity, positive predictive value (PPV), negative predictive value (NPV) and 95% confidence interval (CI) were calculated in both the training and validation groups. ROC analysis was performed using MedCalc software V.18 (Mariakerke, Belgium), while the other analyses used SPSS V.23.0 (IBM company, Chicago, Illinois, USA). ROC curves were drawn using GraphPad Prism version 7.04 (San Diego, California, USA). All tests were two-sided, and a p value less than 0.05 was considered to indicate a significant difference.

## Results

### Comparison of the Imaging Features Among Groups of Cartilaginous Tumors

**Figure 2:**
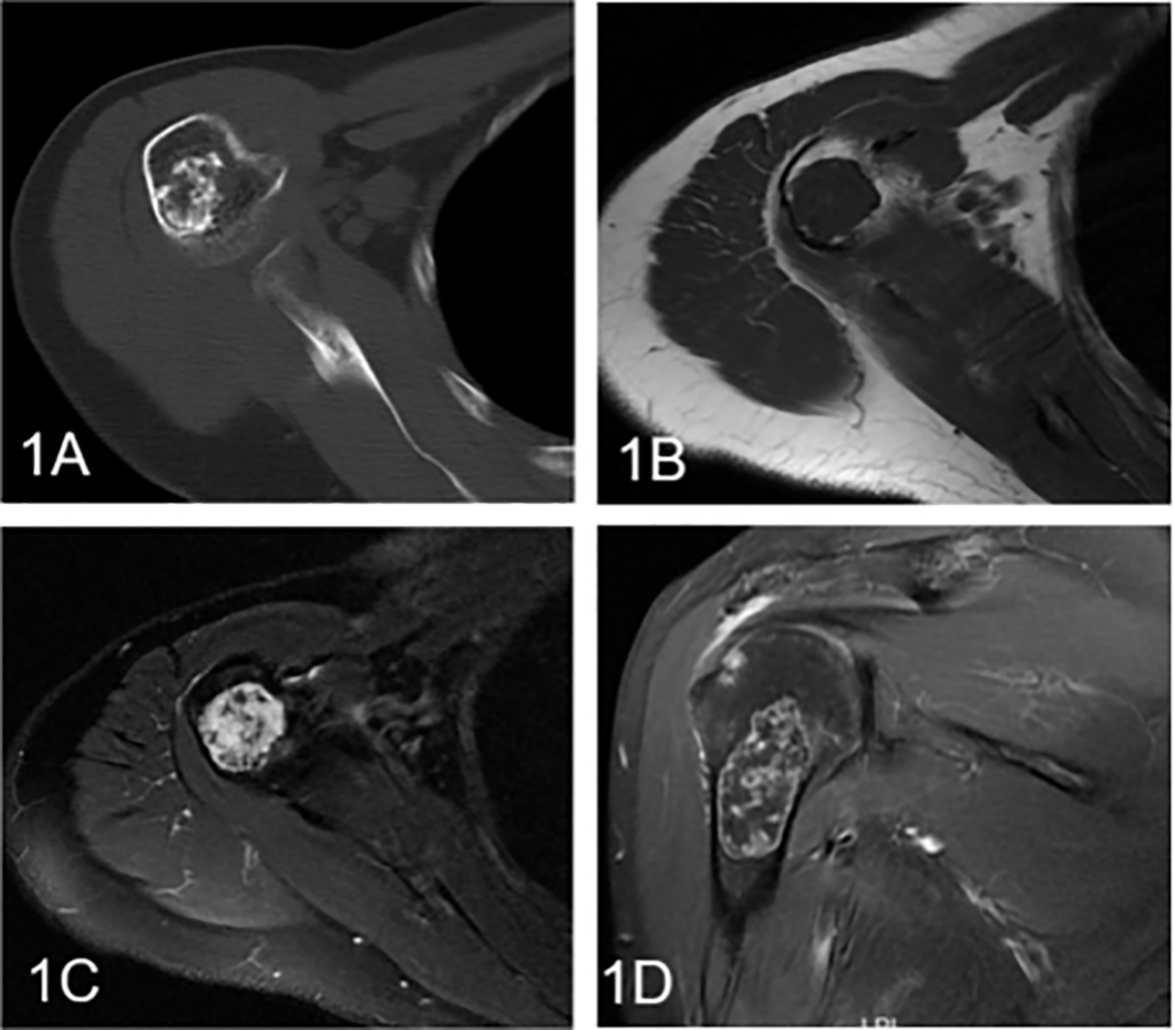
Enchondroma involving the humerus in a 58-year-old female. **(A)** Axial CT shows a well-defined intramedullary osteogenic lesion in the metaphysis of the right humerus, which presents with characteristic chondroid (ring and arc) calcifications. **(B)** Axial T1WI presents a mass with global heterogeneity and a well-defined margin. **(C)** Axial fat-suppressed T2WI shows a heterogeneous hyperintense mass with low signal foci. **(D)** Coronal fat-suppressed contrast-enhanced T1WI demonstrates marginal and septal or ring-and-arc enhancement within the lesion.

**Figure 3:**
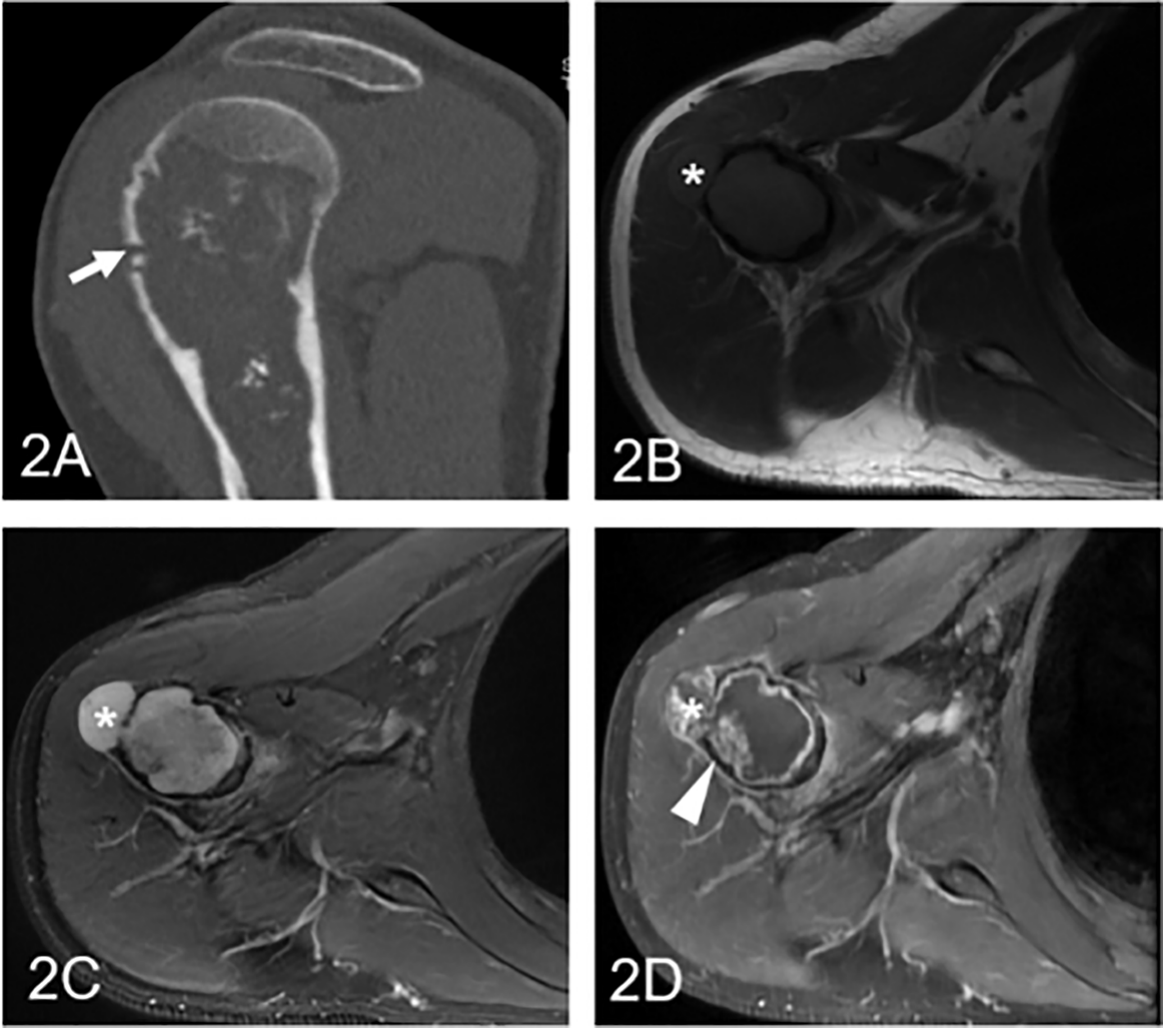
Low-grade chondrosarcoma involving the humerus in a 57-year-old man. **(A)** Coronal CT shows an ill-defined intramedullary osteolytic lesion with several ring and popcorn calcified components, and the arrowhead shows cortical disruption (arrow). **(B)** Axial T1WI presents a homogeneous lesion with an associated soft tissue mass (asterisk). **(C)** Axial fat-suppressed T2WI demonstrates a heterogeneous hyperintense lesion with a soft tissue component (asterisk) and endosteal scalloping of the cortex. **(D)** Coronal fat-suppressed contrast-enhanced T1WI demonstrates patched (arrowhead) and marginal enhancement.

**Figure 4:**
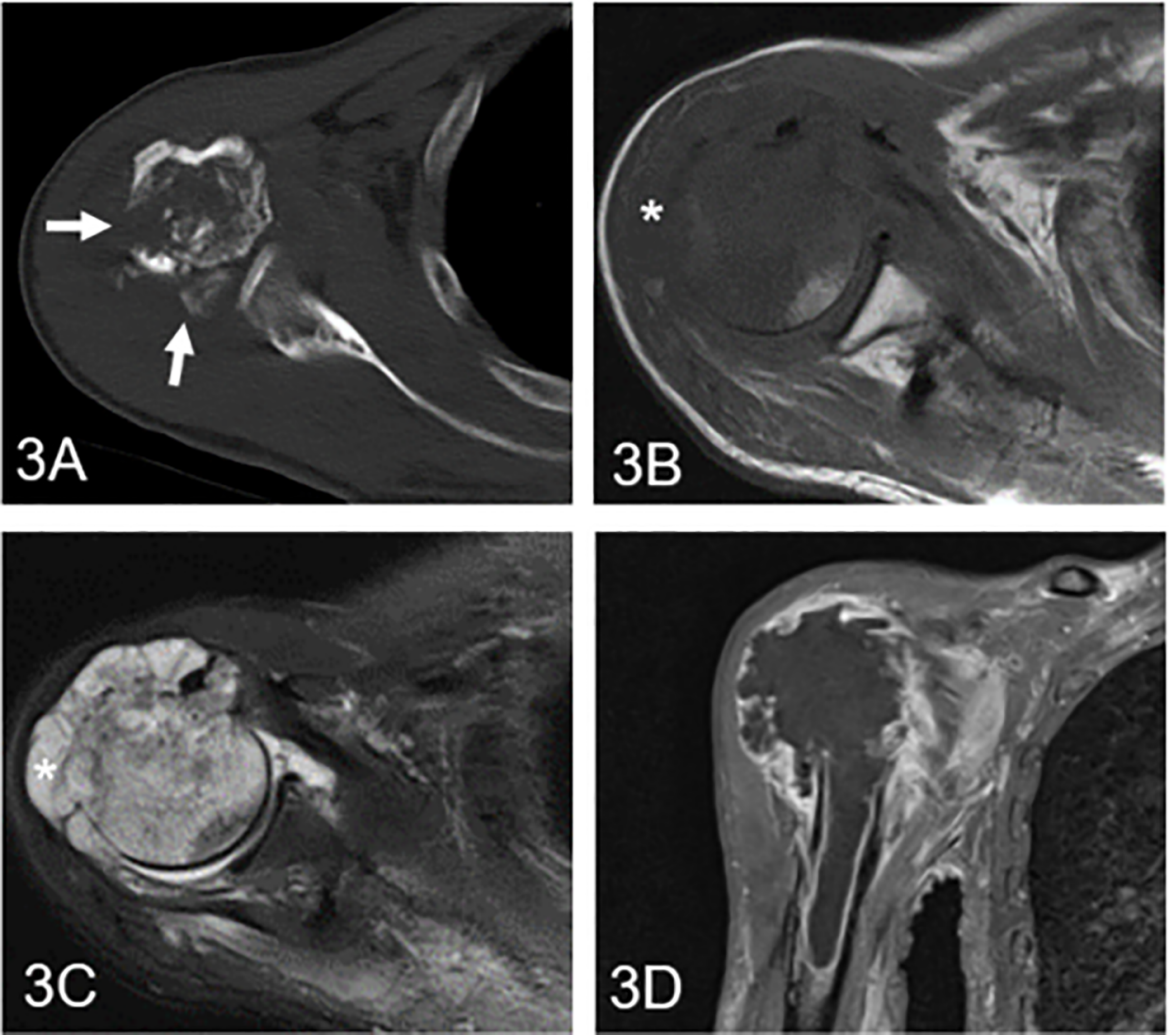
High-grade chondrosarcoma in the proximal humerus in a 61-year-old man. **(A)** Axial CT shows an ill-defined osteolytic lesion with multiple cortical destruction (arrow). **(B)** Axial T1WI presents an irregular soft tissue mass with an aggressive growth pattern (asterisk). **(C)** Axial fat-suppressed T2WI demonstrates a heterogeneous hyperintense mass with low signal components and soft tissue. **(D)** Coronal fat-suppressed contrast-enhanced T1WI shows diffuse and marginal enhancement.

The clinical information and imaging features are summarized in **Table 1**, and a comparison of different subgroups of cartilaginous tumors is also presented. Cartilaginous tumors tended to occur in patients in their fifties and were located in the metaphysis of intramedullary long bones; enchondromas were more likely to occur in females, while chondrosarcomas occurred most often in males. Chondrosarcomas more frequently presented with symptoms and larger diameters than enchondromas (P < 0.05), while there was no difference between low-grade and high-grade chondrosarcomas (P > 0.05). Enchondromas and low-grade chondrosarcomas always included ring and arc or plaque chondroid matrix. The area of calcification decreased with increasing malignancy, while cortical destruction, periosteal reaction and blurry edges increased. Endosteal scalloping of the cortex was more frequent in chondrosarcomas, especially in low-grade chondrosarcomas. Fat replacement was always shown in enchondromas and low-grade chondrosarcomas (P < 0.05), while hemorrhage, peritumoral edema and soft tissue mass were only shown in chondrosarcomas. A soft tissue mass always presented with global hypodensity on CT or heterogeneous hyperintensity on T2WI. For the enhanced pattern, enchondromas all presented with continuous ring- and arc- enhancement of the lesion, while chondrosarcomas were more likely to manifest as a small patchy enhancement with or without ring-and arc- enhancement or a large patchy, nodule enhancement or with obvious enhancement of the internal septa thickness (**Figures 2**–**4**).

**Table 1:** Clinical information and univariate analysis between imaging features and tumor grades.

Variables	Enchondromas (n=29)	Low-grade chondrosarcomas (n=20)	High-grade chondrosarcomas (n=16)	Malignant (n=36)	Benign *vs.* Low grade (P)	Low grade *vs.* High grade (P)	Benign *vs.* Malignant (P)
Age	49 (40–59)	51.5 (39–62)	55.5 (45–62)	54 (44.3–60)	0.662	0.440	0.251
Gender					0.110	0.709	**0.04**
Male	8 (27.6)	10 (50)	9 (56.3)	19 (52.8)			
Female	21 (72.4)	10 (50)	7 (43.8)	17 (47.2)			
Symptomatic	17 (58.6)	19 (95)	12 (75)	31 (86.1)	**0.007**	0.149	**0.012**
Largest diameter/mm	4.1 (2.5–6.5)	6.6 (3.4–18)	7.1 (5.9–10.8)	6.7 (4.5–12.9)	**0.020**	0.440	**<0.001**
Aspect ratio	1.8 (1.5–2.8)	2.5 (1.2–4)	2 (1.2–3.6)	2.2 (1.3–3.5)	0.495	0.962	0.601
Location					0.054	0.103	0.138
Epiphysis	1 (3.4)	4 (20)	3 (18.8)	7 (19.4)			
Metaphysis	21 (72.4)	15 (75)	8 (50)	23 (63.9)			
Diaphysis	7 (24.1)	1 (5)	5 (31.3)	6 (16.7)			
Calcified shape					0.295	0.144	**0.033**
Non calcification	0 (0)	1 (5)	1 (6.3)	2 (5.6)			
Ring and plaque	27 (93.1)	16 (80)	8 (50)	24 (66.7)			
Ground glass	2 (6.9)	3 (15)	7 (43.8)	10 (27.8)			
Calcified area					**0.011**	0.402	**<0.001**
<1/3	6 (20.7)	11 (55)	12 (75)	23 (63.9)			
1/3~2/3	7 (24.1)	6 (30)	2 (12.5)	8 (22.2)			
>2/3	16 (55.2)	3 (15)	2 (12.5)	5 (13.9)			
Endosteal scalloping	2 (6.9)	15 (75)	10 (62.5)	25 (69.4)	**<0.001**	0.418	**<0.001**
Periosteal reaction	0 (0)	8 (40)	11 (68.8)	19 (52.8)	**<0.001**	0.086	**<0.001**
Cortical destruction	0 (0)	9 (45)	13 (81.3)	22 (61.1)	**<0.001**	**0.029**	**<0.001**
Blurry edge	5 (17.2)	10 (50)	11 (68.8)	21 (58.3)	**0.014**	0.257	**0.001**
Fat replacement	20 (69)	8 (40)	0 (0)	8 (22.2)	**0.044**	**0.005**	**<0.001**
Hemorrhage	0 (0)	4 (40)	5 (31.3)	9 (25)	**0.023**	0.470	**0.004**
Peritumoral edema	0 (0)	15 (75)	13 (81.3)	28 (77.8)	**<0.001**	0.709	**<0.001**
Soft tissue mass	0 (0)	6 (30)	10 (62.5)	16 (44.4)	**0.003**	0.051	**<0.001**
Enhanced pattern (n=40)					**<0.001**	0.146	**<0.001**
I type	17 (58.6)	1 (5)	0 (0)	1 (2.8)			
II type	0 (0)	12 (60)	5 (31.3)	17 (47.2)			
III type	0 (0)	1 (5)	4 (25)	5 (13.9)			

The bold values means P < 0.05.

### Different Models to Discriminate the Differentiation of Cartilaginous Tumors

Predictor models based on imaging features, CTTA features, T1WI texture analysis (T1-TA) features and T2WI texture analysis (T2-TA) features are shown in **Table 2**. Regarding imaging features, endosteal scalloping was an independent predictor to differentiate enchondromas from low-grade chondrosarcomas, while cortical destruction could differentiate low-grade chondrosarcomas from high-grade chondrosarcomas. To distinguish benign cartilaginous tumors from malignant cartilaginous tumors, we found that sex, calcified shape and endosteal scalloping might be useful. In terms of texture analysis, we found that the CTTA feature model could differentiate cartilaginous tumors well, while the T1-TA and T2-TA feature models could not differentiate low-grade from high-grade chondrosarcomas. For texture analysis features, variance, mean, perc.01%, perc.10%, perc.99% and kurtosis were extracted through multivariate regression analysis.

**Table 2:** Multivariate analysis among different models.

Model	Compared Tumor Grades	Independent Predictors	Odds Ratio (95%CI)	P
Imaging feature model	Benign *vs.* low-grade	Endosteal scalloping	0.042 (0.004, 0.439)	0.008
	Low-grade *vs.* high-grade	Cortical destruction	5.296 (1.143, 24.548)	0.033
	Benign vs malignant	Gender	0.041 (0.002, 0.813)	0.036
		Calcified shape	205.140 (1.55, 27147.14)	0.033
		Endosteal scalloping	0.007 (0.001, 0.186)	0.003
CTTA feature model	Benign *vs.* low-grade	Variance	1.001 (1.001, 1.002)	0.002
	Low-grade *vs.* high-grade	Mean	1.019 (1.004, 1.034)	0.013
	Benign *vs.* malignant	Variance	1.001 (1.000, 1.001)	0.04
		Perc.01%	0.974 (0.953, 0.995)	0.014
T1-TA feature model	Benign *vs.* low-grade	Kurtosis	1.645 (1.1, 2.459)	0.015
		Perc.10%	0.861 (0.759, 0.997)	0.019
	Benign *vs.* malignant	Kurtosis	1.205 (1.032, 1.407)	0.019
		Perc.10%	0.84 (0.759, 0.930)	0.001
		Perc.99%	1.09 (1.032, 1.152)	0.002
T2-TA feature model	Benign *vs.* low-grade	Variance	1.002 (1.001, 1.003)	0.007
	Benign *vs.* malignant	Variance	1.002 (1.001, 1.003)	0.003

### Diagnostic Performance of Each Model

**Figure 5:**
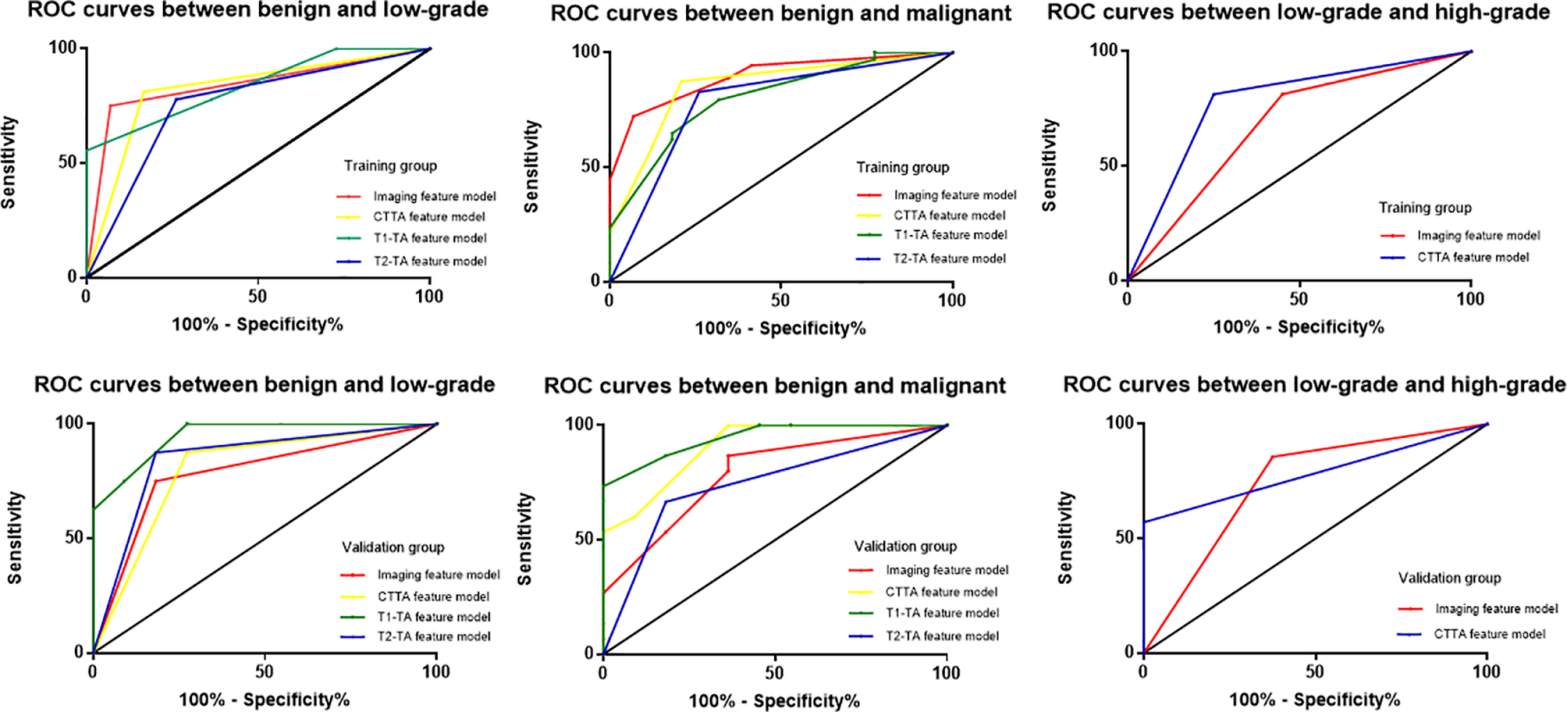
ROC curves among different models to distinguish cartilaginous tumors in both the training group and validation group.

The diagnostic performance of the imaging, CTTA, T1-TA, and T2-TA feature models between the training and validation groups are listed in **Table 3**, and the ROC curves are presented in **Figure 5**. For differentiating benign cartilaginous tumors from low-grade chondrosarcomas, we found that the imaging features model reached the highest accuracy rate (83.7%) and AUC (0.841), with a sensitivity value of 75%, a specificity value of 93.1%, a PPV of 88.2% and NPV of 84.4%, followed by the CTTA and T1-TA feature models (accuracy rate: 80%). The validation group also achieved good performance, with an AUC of 0.784. The CTTA feature model was the best method to distinguish low-grade chondrosarcomas from high-grade chondrosarcomas, with an accuracy rate of 71.9% and 80% in the training and validation groups respectively; T1-TA and T2-TA could not distinguish them well. In terms of differentiating benign cartilaginous tumors from malignant cartilaginous tumors, the imaging feature model performed the best, with an accuracy rate of 89.2% and AUC of 0.896, followed by the T1-TA feature model, with an accuracy rate of 80.4% and AUC of 0.804.

**Table 3:** Analysis efficiency in different models in the training group and validation group.

Model	Group	Compared Tumor Grades	Accuracy rate	Sensitivity	Specificity	PPV	NPV	AUC (95% CI)
Imaging feature model	Training	Benign *vs*. low-grade	83.7	75	93.1	88.2	84.4	0.841 (0.708, 0.929)
		Low-grade *vs*. high-grade	66.7	81.3	55	59.1	78.6	0.681 (0.505, 0.826)
		Benign *vs*. malignant	89.2	72.2	93.1	92.9	73	0.896 (0.795, 0.958)
	Validation	Benign *vs*. low-grade	78.9	75	81.8	75	81.8	0.784 (0.538, 0.936)
		Low-grade *vs*. high-grade	73.3	85.7	62.5	66.7	83.3	0.741 (0.457, 0.926)
		Benign *vs*. malignant	76.9	86.7	63.6	76.5	77.8	0.788 (0.584, 0.922)
CTTA feature model	Training	Benign *vs*. low-grade	80	81.3	83.3	76.5	87	0.823 (0.670, 0.925)
		Low-grade *vs*. high-grade	71.9	81.3	75	76.5	80	0.785 (0.600, 0.907)
		Benign *vs*. malignant	71.4	79.2	87.5	84.8	82.6	0.854 (0.734, 0.934)
	Validation	Benign *vs*. low-grade	78.9	87.5	72.7	70	88.9	0.801 (0.557, 0.946)
		Low-grade *vs*. high-grade	80	57.1	100	100	72.7	0.786 (0.504, 0.950)
		Benign *vs*. malignant	84.6	100	63.6	78.9	100	0.906 (0.726, 0.985)
T1-TA feature model	Training	Benign *vs*. low-grade	80	100	55.6	73.3	100	0.838 (0.688, 0.935)
		Benign *vs*. malignant	80.4	79.4	68.2	79.4	68.2	0.804 (0.676, 0.898)
	Validation	Benign *vs*. low-grade	84.2	100	72.7	72.7	100	0.949 (0.742, 0.999)
		Benign *vs*. malignant	84.6	73.3	100	100	73.3	0.945 (0.780, 0.996)
T2-TA feature model	Training	Benign *vs*. low-grade	75.6	77.8	73.9	70	81	0.758 (0.600, 0.878)
		Benign *vs*. malignant	79.3	82.9	73.9	82.9	73.9	0.796 (0.670, 0.891)
	Validation	Benign *vs*. low-grade	78.9	75	81.8	75	81.8	0.784 (0.538, 0.936)
		Benign *vs*. malignant	84.2	87.5	81.8	77.8	90	0.847 (0.610, 0.968)

PPV, positive predictive value; NPV, negative predictive value.

## Discussion

Accurate grading of cartilaginous tumors is of paramount importance in the management of these lesions, ranging from follow-up for enchondromas to curettage for low-grade chondrosarcomas and amputation or extended resection for high-grade chondrosarcomas (20). In this study, we evaluated the diagnostic performance of CTTA and MRI-based texture analysis for grading cartilaginous tumors in long bones and compared these findings to radiological features both in the training and validation groups. After multivariate analysis, we found that the imaging features model reached the highest accuracy rate both in differentiating enchondromas from low-grade chondrosarcomas and from malignant cartilaginous tumors. Furthermore, the CTTA feature model was the best method to distinguish low-grade chondrosarcomas from high-grade chondrosarcomas, while MRI-based texture analysis could not effectively distinguish low-grade chondrosarcomas from high-grade chondrosarcomas.

In our study, radiological features were evaluated both by MRI and CT, which included calcification information when compared to other studies (17, 21, 22). Imaging features differ when the grade of a cartilaginous tumor changes. We found that sex, calcified shape and endosteal scalloping were independent predictors of the differentiation of benign and malignant cartilaginous tumors. Enchondromas always present with ring and plaque calcification, which indicates mature cartilage matrix differentiation. Douis et al. (23) found that pain related to lesions, tumor length, endosteal scalloping, bone expansion, cortical destruction and soft tissue mass could distinguish enchondromas from low-grade chondrosarcomas, while dynamic contrast-enhanced MRI was not useful in differentiating enchondromas from low-grade chondrosarcomas. Our study also showed that invasive features, such as cortical destruction, blurry edges, periosteal reaction, peritumoral edema and soft tissue mass were more likely to be present in chondrosarcomas, especially in high-grade chondrosarcomas. However, in contrast-enhanced MRI, we found that continuous ring-and-arc enhancement was always shown in enchondromas, while two other types of enhanced patterns were more frequently present in chondrosarcomas. In addition to dynamic contrast-enhanced MRI, diffusion-weighted imaging is another functional sequence used in imaging diagnosis; however, it does not aid in the distinction or grading of cartilaginous tumors (24). Coninck et al. (16) demonstrated that dynamic contrast-enhanced MRI played an important role in distinguishing enchondromas and low-grade chondrosarcomas, with 93.4% accuracy in predicting the diagnosis of chondrosarcomas. When differentiating low-grade and high-grade chondrosarcomas, Sharif et al. (25) found that soft tissue mass, periosteal reaction and bone edema suggested high-grade chondrosarcomas, which was consistent with our results. They also found that soft tissue mass and cortical destruction were predictors of high-grade chondrosarcomas in long bones. Soft tissue mass can be found in 28% of chondrosarcomas and can replace marrow fat, indicating a pathognomonic sign of malignancy (21).

The most compelling result is that CTTA could distinguish low-grade from high-grade chondrosarcomas, while MRI-based texture analysis was not able to show the correlation. This is especially beyond the expectation that CT performs better than MRI, which may be because thin-slice CT imaging demonstrates calcification well within the lesion. Most of the low-grade chondrosarcomas presented with ring and plaque calcification, while nearly half of the high-grade chondrosarcomas showed ground glass calcification due to an immature cartilage matrix. Lisson et al. (3) included 11 patients with enchondromas and 11 patients with low-grade chondrosarcomas, and only four texture analyses were obtained for each lesion. The authors found that kurtosis in contrast-enhanced T1WI had the greatest power of discrimination, with 86% accuracy. In our study, we found that kurtosis extracted from non-enhanced T1WI was an independent predictor to differentiate benign cartilaginous tumors from chondrosarcomas. Yin et al. (26) used a clinical radiomics nomogram combined with clinical characteristics to successfully predict the early recurrence of pelvic chondrosarcomas and found that a radiomics model based on T1WI outperformed than T2WI or contrast-enhanced radiomics to predict recurrence.

Many texture analysis features based on CT or MRI have been published and suggested depending on histogram analysis or other matrixes (27, 28). We evaluated 9 histogram features and found that variance, mean, perc.01%, perc.10%, perc.99% and kurtosis had good discriminatory power to grade cartilaginous tumors in different feature models. Hu et al. (29) extracted 22 histogram parameters and integrated them with CT morphological features, which can be used to predict lung metastasis in patients with colorectal cancer. Histogram features may be related to the tumor microenvironment. For example, variance measures the deviation of gray levels from the mean and represents the extent of the histogram, which may reflect on morphologic imaging performance (4). CT and MRI for imaging features should always be initially considered; if there is a need for differentiation of malignant tumor grading, CTTA should be performed.

Our study has several limitations. First, due to our retrospective study design, there may have been inherent selection and unavoidable biases. Second, several MRI and CT machines and protocols have been used, and although we had standardized images before analysis, possible bias may still have been introduced. Third, the number of patients was relatively small, and the number of extracted features was also small; thus, further studies with larger samples or numerous features are needed. Fourth, we did not combine imaging feature models with a texture analysis feature model because both CT and MRI imaging features were incorporated into the imaging feature model; thus, it was impossible to combine an imaging feature model with CT or MRI alone. Finally, we did not use dynamic contrast-enhanced MRI images; on the one hand, not all patients had undergone enhanced MRI, and on the other hand, a recent study found that contrast-enhanced MRI may not distinguish enchondromas from low-grade chondrosarcomas (23).

In conclusion, the imaging feature model and CTTA- or MRI-based texture analysis have the potential to differentiate the grade of cartilaginous tumors in long bones. MRI-based texture analysis failed to grade chondrosarcomas.

## Data Availability Statement

The original contributions presented in the study are included in the article/supplementary material. Further inquiries can be directed to the corresponding authors.

## Author Contributions

X-YD and H-YC completed manuscript together. J-NY collected validation group data. J-YC and X-LZ processed the data and the statistics. G-LS and R-SY gave the support of everything we need. All authors did literature researches. All authors contributed to the article and approved the submitted version.

## Funding

This work was reported by Zhejiang Province Medical, Science and Technology Project, the number is 2021KY091.

## Conflict of Interest

The authors declare that the research was conducted in the absence of any commercial or financial relationships that could be construed as a potential conflict of interest.

## Publisher’s Note

All claims expressed in this article are solely those of the authors and do not necessarily represent those of their affiliated organizations, or those of the publisher, the editors and the reviewers. Any product that may be evaluated in this article, or claim that may be made by its manufacturer, is not guaranteed or endorsed by the publisher.

## Abbreviations

CTTA, Computed Tomography Texture Analysis; T1-TA, T1-weighted imaging Texture Analysis; T2-TA, T2-weighted imaging Texture; ROI, Region of Interest; ROC, Receiver Operating Characteristic; PPV, Positive predictive value; NPV, Negative predictive value; CI, Confidence Interval.
